# Demographic and prognostic factors affecting survival in acute myelomonocytic leukemia with abnormal eosinophils

**DOI:** 10.1016/j.htct.2026.106463

**Published:** 2026-05-13

**Authors:** Beau Hsia, Jasika Grover, Mahadevan Iyer, Assal Sadighian, Anton Sachs, Bhanu Upadhyayula, Timothy J. Brown

**Affiliations:** Creighton University Phoenix Health Sciences Campus, Phoenix, AZ, USA

**Keywords:** National cancer database, Prognostic factors, Insurance status, Socioeconomic factors, Overall survival, Age, Race, Comorbidities, Acute myeloid leukemia

## Abstract

**Introduction:**

Acute myelomonocytic leukemia with abnormal eosinophils is an extremely rare, malignant acute myeloid leukemia subtype. Using the National Cancer Database, this study evaluated the demographic and prognostic factors affecting the overall survival and mortality rates of patients. To the extent of our knowledge, this is the most comprehensive study to date on the prognostic and socioeconomic factors of this leukemia subgroup.

**Methods:**

Patients with acute myelomonocytic leukemia with abnormal eosinophils were identified in the National Cancer Database between 2004 and 2020 using the ICD-O-3 histology code 9871. Time-to-event outcomes were analyzed by the Kaplan-Meier method, log-rank tests, and multivariable Cox hazard regression models to understand the association of demographic and prognostic factors on survival.

**Results:**

A total of 2138 patients were identified. Patients with a Charlson-Deyo score of 2 or 3 had a 91% (Hazard Ratio = 1.91; 95% Confidence Interval: 1.52–2.39; p-value ≤0.001) and 79% (Hazard Ratio = 1.79; 95% Confidence Interval: 1.34–2.40; p-value ≤0.001) increased mortality risk, respectively, compared to those with a score of 0. Compared to patients with private insurance/managed care, those with other government insurance, Medicaid, and Medicare had a 46% (Hazard Ratio = 1.46; 95% Confidence Interval: 0.87–2.45; p-value ≤0.156), 57% (Hazard Ratio = 1.57; 95% Confidence Interval: 1.26–1.97; p-value ≤0.001), and 32% (Hazard Ratio = 1.32; 95% Confidence Interval: 1.11–1.58; p-value ≤0.001) greater mortality risk, respectively.

**Conclusion:**

Generally, older patients with comorbidities who did not have private insurance had poorer overall survival. No significant difference in overall survival was associated with median household income quartiles.

## Introduction

Acute myelomonocytic leukemia with abnormal eosinophils (AMMLEo) is an extremely rare, malignant acute myeloid leukemia (AML) subtype. Acute myelomonocytic leukemia (AMML) is a type of AML, a group of blood cancers marked by a rapid increase in the white blood cell count [1]. The most common cytogenetic changes in this form of leukemia include inv(16)(p13q22) or the variant t(16;16)(p13;q22). AML with inv(16)(p13q22), is commonly seen in young patients and typically exhibits both granulocytic and monocytic differentiation with abnormal eosinophils [2].

AML itself makes up about 15–20 % of all acute leukemia cases and according to Surveillance, Epidemiology, and End Results (SEER) program data, AMMLEo accounts for 5–8 % of all AML cases. AMMoL originates within the bone marrow and peripheral blood; while the disease manifests across a broad age spectrum, it is most frequently diagnosed in pediatric and young adult populations [3].

Chemotherapy is the standard treatment for AMMLEo [1,4]. AMMLEo presents with a documented five-year survival rate of around 51.7 %, regardless of grade. Survival analysis revealed that individuals with eosinophilia had an overall survival rate of 64 % at 36 months, with a median survival time of 23.3 months. Conversely, patients without eosinophilia exhibited a lower overall survival rate of 38 %, with a median survival time of 15.8 months [5]. Understanding the variables affecting prognosis holds significance for patients and healthcare providers. However, due to its rarity, there is a lack of comprehensive studies on factors influencing overall survival. Prior investigations have indicated that factors such as older age, complex cytogenetic abnormalities, and specific genetic mutations correlate with reduced overall survival, with the presence of certain chromosomal rearrangements associated with poor prognosis and more aggressive disease [6,7].

This study aims to comprehensively expand on factors influencing the prognosis of AMMLEo. An emphasis is placed on demographics including age, sex, race, income, and education as well as clinical aspects such as tumor size, analytical stage, neoadjuvant chemotherapy, neoadjuvant radiation, distance traveled for health care, and Charlson-Deyo (CD) comorbidity score. The goal is to offer a thorough understanding of how these factors collectively affect overall survival in individuals with this type of leukemia.

## Materials and methods

This is a retrospective cohort study of patients diagnosed with AMMLEo from 2004 to 2020 and registered in the National Cancer Database (NCDB). The NCDB is a clinical oncology database derived from hospital registry data of >1500 Commission on Cancer-accredited facilities with information on patient characteristics, tumor staging, tumor histology, type of first treatment, disease recurrence, and survival. This patient data was made accessible to the authors through the Participant User Data Files program.

Patients with AMMLEo were identified from NCDB data using the ICD-O-3 histology code 9871, which classifies tumors based on their morphological and histological features. Patients were then selected if they had behavior code 3 (invasive). Patients were excluded from the cohort if they had any missing clinical or demographic data or if they had concurrent tumors.

### Covariates

Patients were analyzed by age, sex, race, education, income, insurance status, tumor size, analytical stage, CD comorbidity score, primary anatomic site, primary radiation, primary and adjuvant chemotherapy, and distance traveled for health care.

Race was categorized into three groups: White, African-American, and Other. The race group categorized as other includes American Indian, Aleutian or Eskimo, Chinese, Japanese, Filipino, Hawaiian, Korean, Vietnamese, Kampuchean, Asian Indian or Pakistani not otherwise specified (NOS), Asian Indian, Micronesian NOS, Other Asian, Asian NOS, Oriental NOS, and Pacific Islander NOS.

Income was measured by median household income from 2016 to 2020 for the ZIP code where the patient resided at the time of diagnosis. Education was measured in 2020 by the percentage of residents within the patient’s ZIP code of residence who did not graduate from high school.

The staging was measured by the NCDB analytical stage. Clinical staging was used when the variable NCDB analytical stage was not available. Insurance status was categorized into five groups: uninsured, private, Medicare, Medicaid, and other government insurance.

Distance traveled for health care was measured by the miles between the patient’s residence and the hospital that reported the case. The CD score, which measures comorbidities, was used to classify patients into groups with scores of 0, 1, 2, and ≥3 [8].

The primary outcome of interest was overall survival, defined from the date of diagnosis until the date of death, censored at the date of last contact in the database. Independent prognostic factors were identified using a multivariable Cox hazard regression model. Kaplan-Meier curves were plotted and the overall survival of the variables of interest was measured at two, five, and ten years using survival tables. Variables included in the multivariable Cox model were age, sex, race, insurance status, education, income, CD comorbidity score, analytic stage, primary anatomic site, primary radiation, and primary and adjuvant chemotherapy, all of which were a priori variables of interest. Patients within the same facility were accounted for using a robust sandwich covariance matrix. LOESS methods were employed to examine the functional form of continuous variables, while log-log survival curves and the inclusion of time-dependent covariates were utilized to evaluate the proportional hazards assumption for each variable.

### Statistical considerations

The descriptive statistics, unadjusted survival analysis, and multivariable analysis for this study were conducted using the IBM Statistical Package for the Social Sciences (SPSS) version 27 (IBM Corp., Armonk, NY). Patients with any missing clinical or demographic data were excluded from the cohort. Bonferroni correction was used to adjust the p-value threshold for multiple comparisons, ensuring the family-wise error rate remained controlled at α = 0.05. The University of Arizona Biomedical Institutional Review Board (IRB) reviewed this study (IRB Submission ID: STUDY00003534, Approval No 2001,750–01) and determined that it does not involve human subjects research as defined by the Department of Health and Human Services (DHHS) and Food and Drug Administration (FDA) regulations. Consequently, IRB approval and ongoing review were not required.

## Results

After applying inclusion and exclusion criteria, a total of 2138 patients were included in this analysis after excluding 531 patients due to missing data. Table 1 lists the demographics and clinical factors of the cohort.

**Table 1:** Demographics of the subjects.

Variable	*n* = 2138 (100 %)
**Sex** – n ( %)	
Male	1185 (55.5)
Female	952 (44.4)
**Race** – n ( %)	
White	1847 (86.4)
Black	164 (7.7)
Other	127 (5.9)
**Age (years)**	
Mean ± Standard Deviation	51.38 ± 0.415
Median (Interquartile Range	53.00 (38.5–67.5)
**ZIP Code-Level Median Household Income (2016–2020, $)** – n ( %)	
<$46,277	380 (17.8)
$46,277-$57,856	496 (23.2)
$57,857-$74,062	513 (24)
≥$74,063	749 (35)
**ZIP Code-Level Education (high-school degree, 2020)** – n ( %)	
≥15.3 %	475 (22.2)
9.1 %−15.2 %	598 (28)
5 %−9 %	597 (27.9)
<5 %	468 (21.9)
**Insurance Status** – n ( %)	
Uninsured	91 (4.3)
Private	1085 (50.7)
Medicaid	286 (13.4)
Medicare	644 (30.1)
Other Government	32 (1.5)
**Distance Traveled for healthcare (Miles)**	
Mean ± Standard Deviation	37.474 ± 1.678
Median (Interquartile Range)	17.2 (0–35.25 Miles)
**Charlson-Deyo comorbidity score** – n ( %)	
0	1578 (73.8)
1	362 (16.9)
2	120 (5.6)
>3	78 (3.6)

This cohort was slightly male-dominant (55.5 %) with an overwhelming majority of patients being White (86.4 %). The primary payor of diagnosis for patients was private insurance/managed care (50.7 %) followed by Medicare (30.1 %), which was followed by Medicaid (13.4 %). Patients were mostly spread among income brackets with the slight majority of patients earning more than $74,063 (35.0 %).

**Fig. 1:**
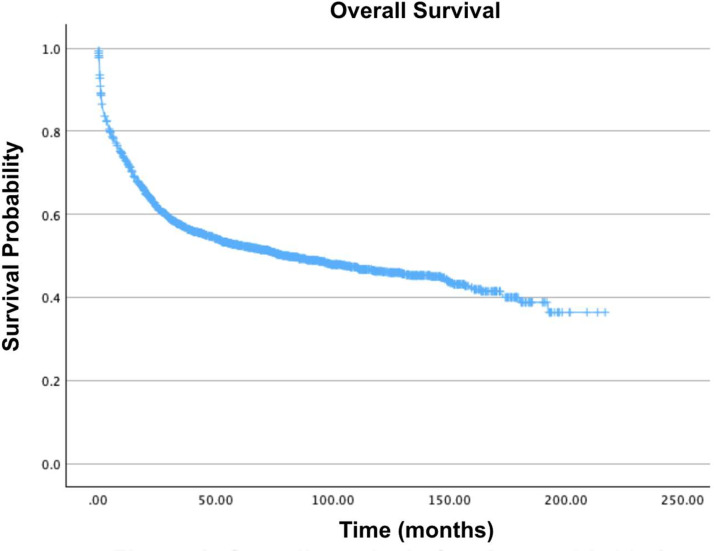
Overall survival of patients with acute myelomonocytic leukemia with abnormal eosinophils.

**Fig. 2:**
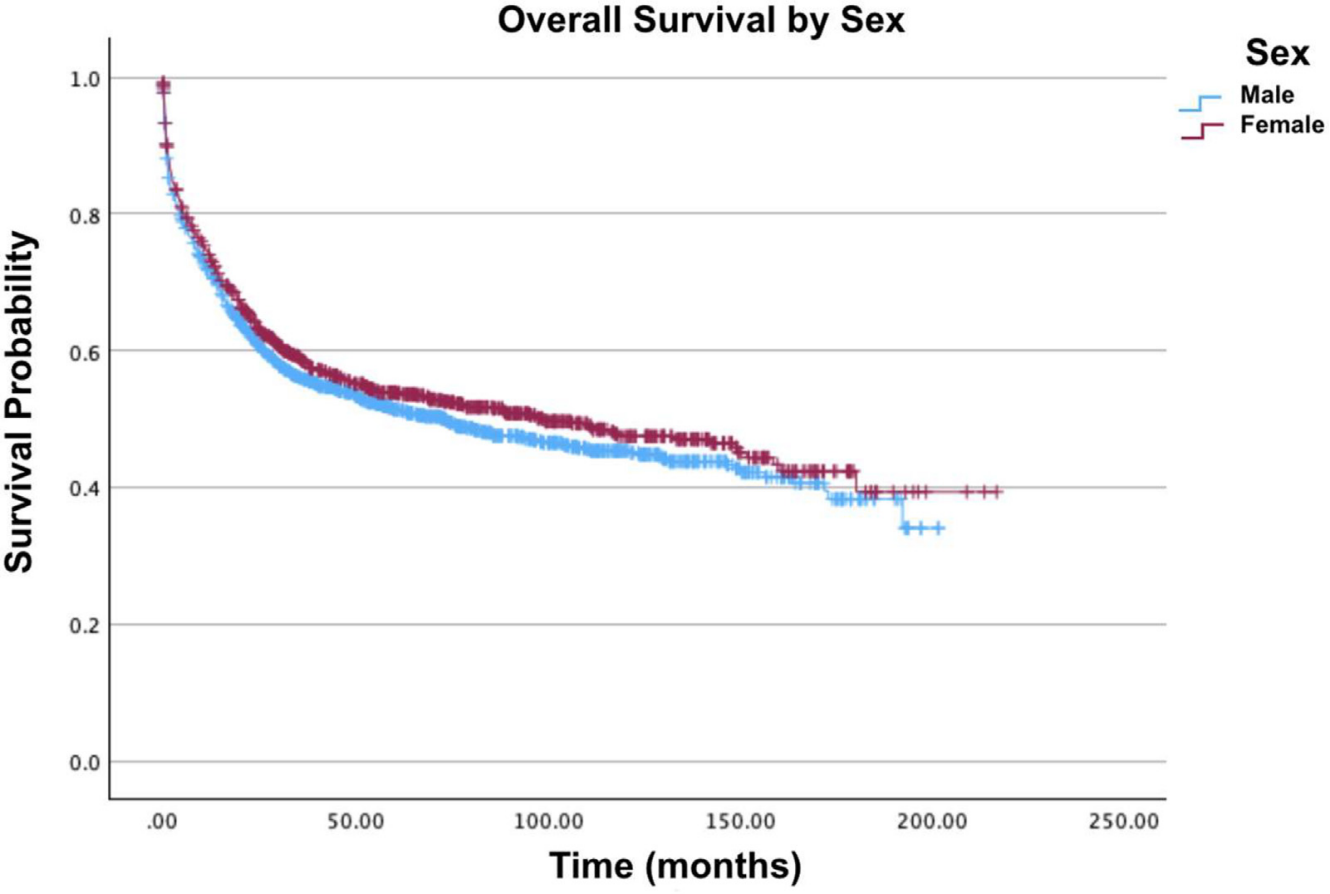
Overall survival by sex of patients with acute myelomonocytic leukemia with abnormal eosinophils.

**Fig. 3:**
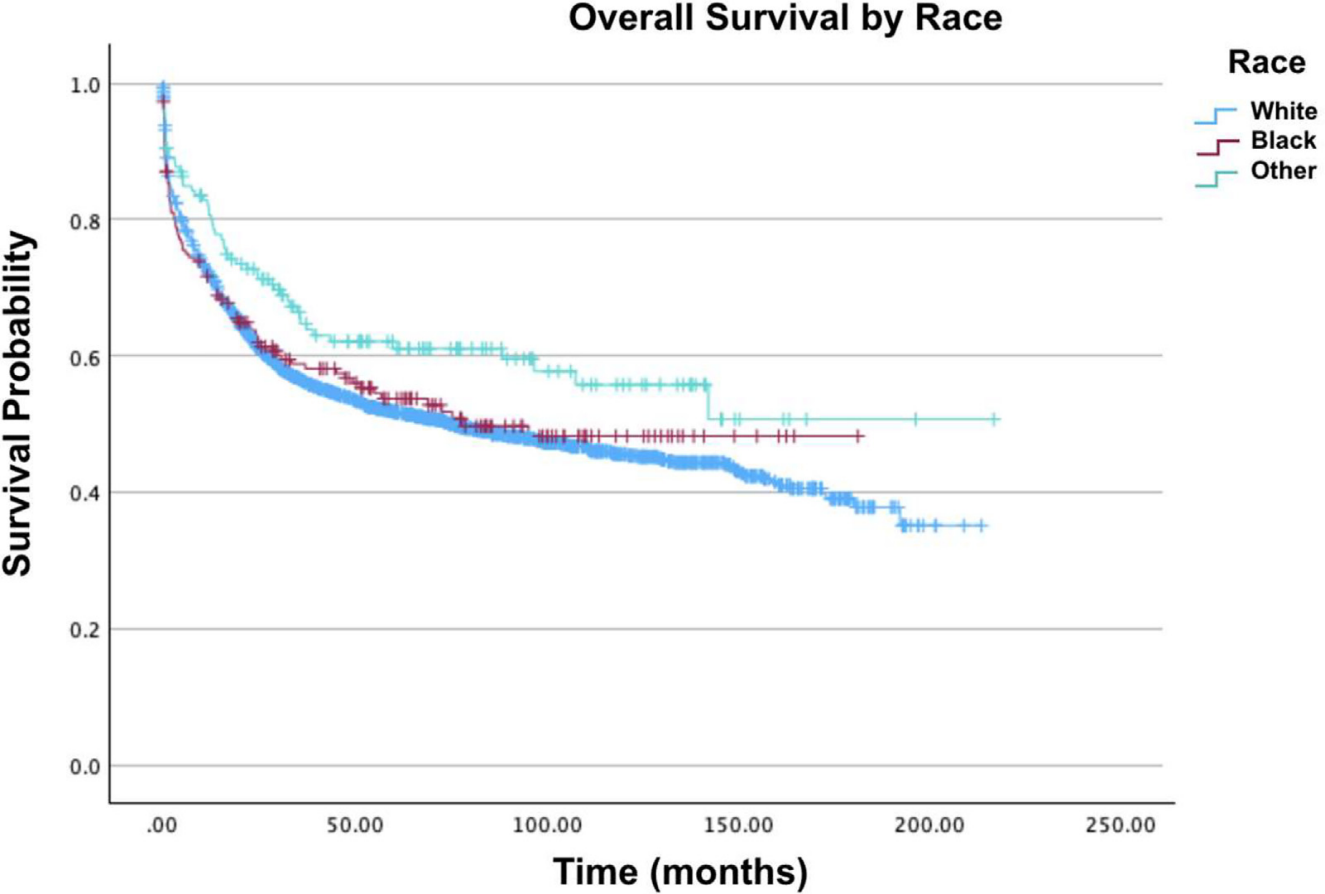
Overall survival by race of patients with acute myelomonocytic leukemia with abnormal eosinophils.

**Fig. 4:**
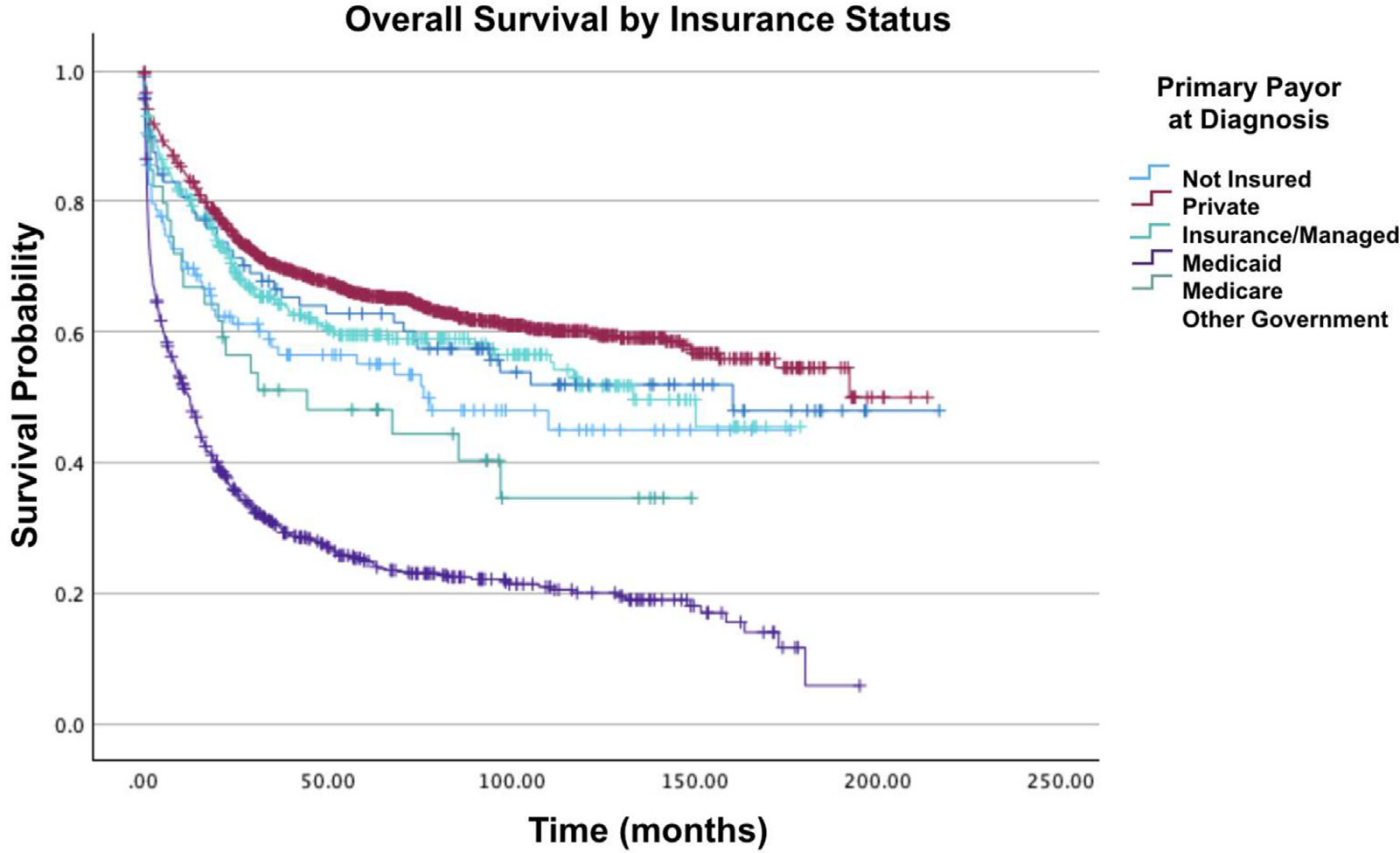
Overall survival by insurance status of patients with acute myelomonocytic leukemia with abnormal eosinophils.

**Fig. 5:**
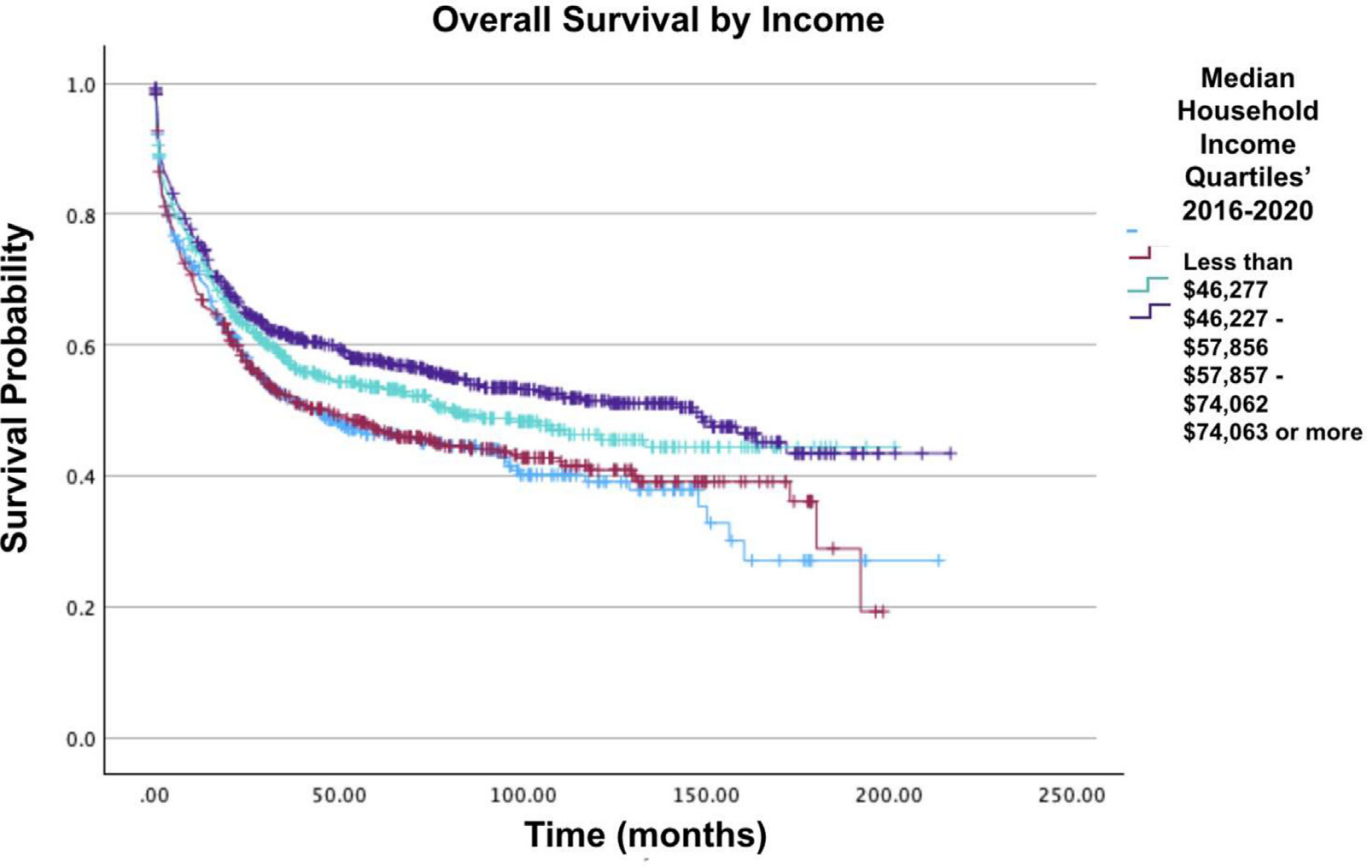
Overall Survival by income of patients with acute myelomonocytic leukemia with abnormal eosinophils.

**Fig. 6:**
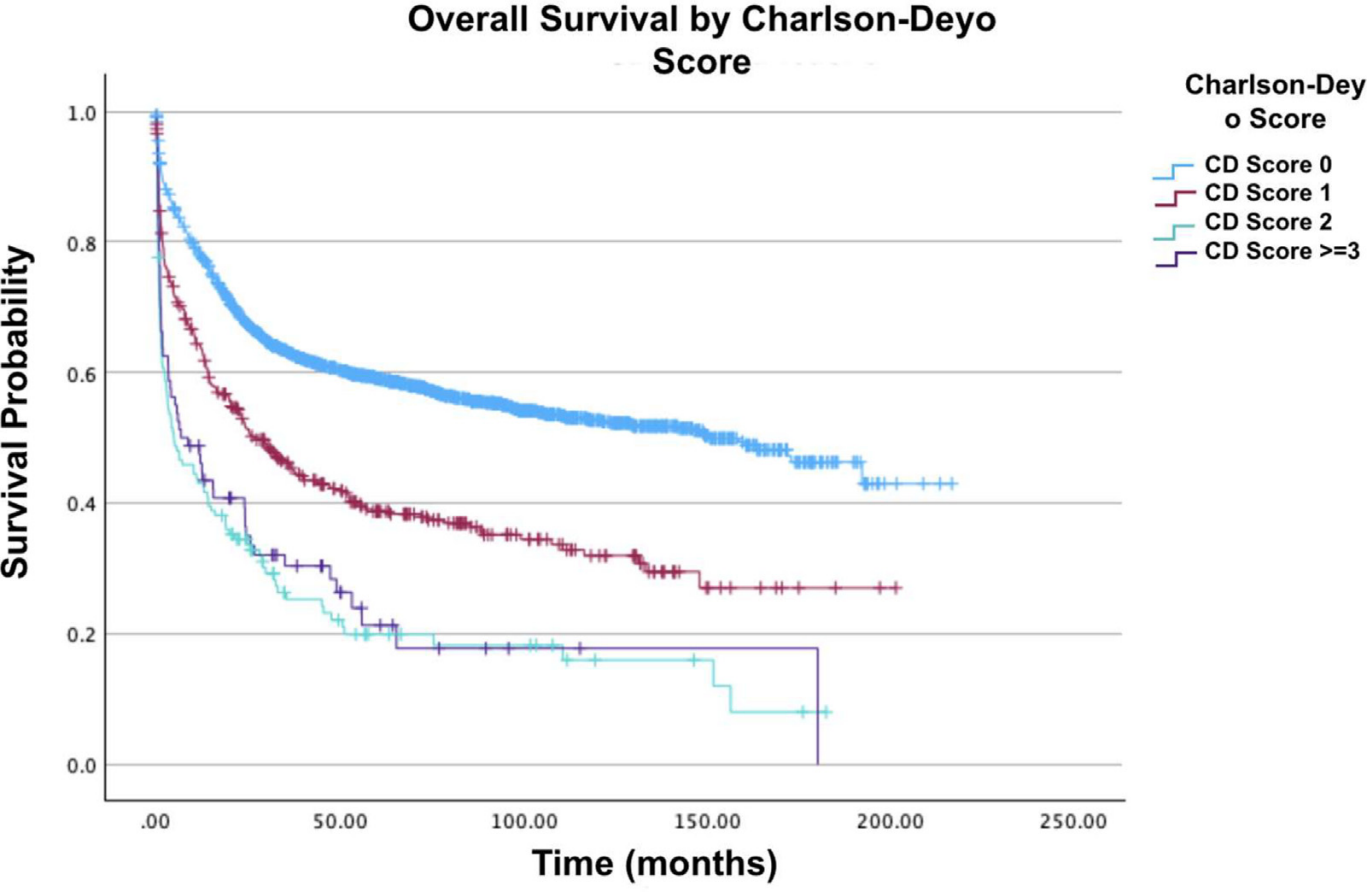
Overall survival by Charlson-Deyo of patients with acute myelomonocytic leukemia with abnormal eosinophils.

The majority of patients (51.1 %) were located in metro area counties with a population exceeding 1 million people. Furthermore, the average age at diagnosis was 51.4 years (standard deviation = 19.2; range: 0–90 years). The overwhelming majority of patients (73.8 %) had a CD score of 0, while 362 patients (16.9 %) had a CD score of 1. Fig. 1, Fig. 2, Fig. 3, Fig. 4, Fig. 5, Fig. 6 show the Kaplan-Meier survival curves of patient cohorts separated by various demographic and clinical groups.

In this cohort, the most common primary site was the bone marrow in 2130 cases (99.6 %). Regarding management, none of the patients underwent primary site surgery. The majority of the cohort received chemotherapy as their primary treatment modality (90.6 %), while 5.7 % received immunotherapy and 3.6 % underwent radiation therapy.

In the present analysis, older age was associated with decreased survival since the oldest age group of 76–100 years had the lowest survival rates at 12 % for two-year survival and 5.5 % for five-year survival. ‘Other races’ exhibited the highest two-year (77.1 %), five-year (64.6 %), and ten-year survival rates (57.9 %) and showed a 34 % decrease in mortality compared to Whites (Hazard Ratio [HR] = 0.66; 95 % Confidence interval [95 % CI]: 0.48–0.90; p-value = 0.009). Univariate analysis revealed that African-Americans had the lowest survival rates compared to Whites and other races (p-value <0.001). However, after multivariate adjustment, this difference was not statistically significant (HR = 0.82; 95 % CI: 0.64–1.06; p-value = 0.135). Females demonstrated better survival in univariate analysis (p-value = 0.003), but this advantage was not significant after multivariate adjustment, showing a paradoxical increase in mortality compared to males (HR = 1.03; 95 % CI: 0.91–1.17; p-value = 0.642).

In analyzing CD scores, patients with a score of 2 or 3 had a 91 % (HR = 1.91; 95 % CI: 1.52–2.39; p-value <0.001) and 79 % (HR = 1.79; 95 % CI: 1.34–2.40; p-value <0.001) increased mortality when compared to those with a score of 0. Regarding insurance status, patients with private insurance/managed care had the greatest two-year, five-year and ten-year survival rates compared to those with Medicare, who had the lowest. Furthermore, compared to patients with private insurance/managed care, those with Medicaid had a 57 % greater mortality risk (HR = 1.57; 95 % CI: 1.26–1.97; p-value <0.001), followed by patients with Medicare who had a 32 % greater mortality risk (HR = 1.32; 95 % CI: 1.11–1.58; p-value <0.001). On comparing levels of income, there was a 26 % decrease in mortality for median household incomes ≥$74,063 compared to median household incomes of $46,227-$57,856 (HR = 0.74; 95 % CI: 0.61–0.90; p-value = 0.002).

Regarding the primary anatomic site, after adjusting for all else, a primary site of bone marrow had increased mortality when compared to all other sites (HR = 0.38; 95 % CI: 0.16- 0.86; p-value = 0.021). In an analysis of treatment modalities, primary chemotherapy demonstrated a 70 % reduction in mortality (HR = 0.30; 95 % CI: 0.25–0.37; p-value < 0.001), while primary immunological treatment showed no statistically significant effect on mortality (HR = 0.83; 95 % CI: 0.58–1.18; p-value = 0.303).

No significant difference in overall survival was associated with education level. Moreover, the quartile with fewer than 5 % of individuals lacking a high school diploma demonstrated the highest two-year, five-year, and ten-year survival rates. In the NCDB data, there were no significant mortality differences associated between patients with CD scores of 2 and ≥3. AJCC staging was not applicable in this cohort Table 2.

**Table 2:** Confidence intervals of various demographics.

Variable	HR (95 % CI)	p-values
Age (5 years)	1.18 (1.15–1.22)	<0.001
Males versus Females	1.03 (0.91–1.17)	0.642
**Race and Ethnicity**		
White versus Black	0.82 (0.64–1.06)	0.135
White versus Other	0.66 (0.48–0.90)	0.009
Black versus Other	0.80 (0.54–1.19)	0.27
**Charlson-Deyo Score**		
0 versus 1	1.29 (1.10–1.51)	0.002
0 versus 2	1.91 (1.52–2.39)	<0.001
0 versus ≥ 3	1.79 (1.34–2.40)	<0.001
1 versus 2	1.48 (1.15–1.91)	0.002
1 versus ≥ 3	1.40 (1.02–1.90)	0.036
2 versus ≥ 3	0.94 (0.66–1.33)	0.728
**ZIP code-level median household income (2020 US Dollars**		
<$46,277 versus $46,227-$57,856	1.09 (0.90–1.33)	0.376
<$46,277 versus $57,867-$74,062	0.91 (0.73–1.13)	0.381
<$46,277 versus ≥$74,063	0.81 (0.65–1.02)	0.072
$46,227-$57,856 versus $57,857-$74,062	0.83 (0.69–1.00)	0.047
$46,227-$57,856 versus ≥$74,063	0.76 (0.61–0.90)	0.002
$57,857-$74,062 versus ≥$74,063	0.89 (0.74–1.07)	0.229
**Insurance**		
None versus Private	0.66 (0.47–0.92)	0.014
None versus Medicaid	1.03 (0.71–1.50)	0.867
None versus Medicare	0.87 (0.61–1.24)	0.44
None versus Other Government	0.95 (0.52–1.75)	0.881
Private versus Medicaid	1.57 (1.26–1.97)	<0.001
Private versus Medicare	1.32 (1.11–1.58)	0.001
Private versus Other Government	1.46 (0.87–2.45)	0.156
Medicaid versus Medicare	0.84 (0.65–1.09)	0.196
Medicaid versus Other Government	0.93 (0.54–1.60)	0.779
Medicare versus Other Government	1.10 (0.65–1.86)	0.727
No chemotherapy versus yes Chemotherapy	0.36 (0.32–0.40)	0.01

HR: Hazard Ratio; 95 % CI: 95 % Confidence interval.

## Discussion

Research on AMMLEo has been limited due to its rarity. However, this study represents one of the largest to examine prognostic factors for AML and is notable for highlighting the significance of the primary anatomic site. The descriptive statistics of this cohort align with the previously reported characteristics of AML patients with abnormal marrow eosinophils. Notably, males have a slightly higher likelihood of developing this form of AML compared to females, consistent with other studies indicating a mild male predominance [7,9,10]. Nonetheless, some reports suggest an equal incidence between the sexes, implying that any actual difference in incidence is likely minimal [11].

The NCDB gathers and analyzes this information to better understand patient prognosis. Although our analysis does not distinguish between AMMLEo-specific mortality and overall mortality, the strong correlation between cancer stage and mortality suggests that patients likely succumbed to cancer-related causes. This underscores the importance of early detection and diagnosis of AMMLEo, as patients diagnosed at an earlier stage and with smaller tumors generally have better survival rates.

AMMLEo is a rare subtype of this hematologic cancer, characterized by the presence of atypical eosinophils in the peripheral blood or bone marrow. Chemotherapy remains the primary treatment, with specific regimens tailored to individual patient factors such as age, overall health, and genetic mutations of leukemic cells. Standard chemotherapy induction regimens for AML typically comprise cytarabine in combination with an anthracycline, such as daunorubicin or idarubicin [7,12]. For patients with actionable mutations, personalized approaches include targeted therapies such as FLT3 inhibitors (e.g., midostaurin) for FLT3-ITD variants, and IDH1/IDH2 inhibitors (e.g., ivosidenib or enasidenib) for IDH1- or IDH2-mutated disease [12,13]. Additionally, stem cell transplantation, either autologous or allogeneic, is considered for patients with high-risk disease or those achieving remission after initial chemotherapy [14].

In cases of AMMLEo, patients with Medicaid or no insurance have a 99 % higher mortality risk compared to those with private insurance [7]. High-income patients show a significant survival benefit over lower-income patients. Additionally, more patients with Medicaid or no insurance are treated at community cancer centers (9.3 % and 6.3 %, respectively) compared to those with Medicare or private insurance (4.2 % and 3.2 %, respectively). Facility type correlates with insurance status, with community cancer centers generally having poorer overall survival rates compared to academic/research programs, comprehensive community cancer programs, or integrated network cancer programs. Facility volume influences outcomes, with higher volume centers being potentially associated with reduced mortality risk [15,16]. Community cancer programs usually encounter anywhere from 100 to 500 newly diagnosed cancer cases annually. In contrast, comprehensive community cancer programs and academic institutions deal with over 500 cases per year. Additional investigations are necessary to grasp the connections between insurance coverage, facility types, facility workload, and overall survival rates. This deeper understanding could enhance patient referrals and accessibility to specific facilities.

One study found that for patients under 60 years old with inv(16) or t(16;16) chromosomal abnormalities had complete remission rates of 92 % and ten-year survival rates of 55 %, whereas patients with a normal karyotype had slightly lower rates of 90 % and 38 %, respectively. Additionally, patients with t(8;21) experienced a complete response rate of 97 % and a ten-year overall survival rate of 61 %. The presence of eosinophilia may lead to even better rates of remission and survival [17].

In cases of AMMLEo, individuals with Medicaid encounter a 57 % higher risk of mortality compared to those with private insurance. Medicare patients face a 32 % elevated risk, while other government insurance holders experience a 46 % increased risk compared to privately insured patients. Moreover, patients in the highest income category demonstrated a notable survival advantage over those in lower-income brackets. These findings align with existing literature, including a Danish study showing low socioeconomic status negatively impacted AML survival (HR = 1.49; 95 % CI 1.25–1.76) [18], and prior research revealing 2.4 times higher mortality in pediatric AML patients from poorer neighborhoods [19].

The five-year survival rate of 51.7 % for patients with AMMLEo reported in this analysis is notable. However, the ten-year survival rate of 45.5 % falls short of the 61 % reported in a study of 5876 young adult patients treated in the United Kingdom Medical Research Council Trials. One potential factor contributing to this difference could be the selection criteria, which included all candidates receiving chemotherapy, immunological treatment, and radiation as their primary form of therapy [20].

The present study is not without limitations. As a retrospective cohort study, it is subject to the inherent limitations of this design, including the lack of granularity that could be achieved with a prospective study. While retrospective analyses are valuable for exploring large datasets, they are constrained by pre-existing data collection practices. For instance, we were unable to capture more nuanced clinical details that may influence outcomes, such as detailed treatment responses or additional comorbidities not registered in the NCDB. Moreover, relying solely on variables obtained from the NCDB and contending with missing data within our cohort limits the breadth of the analysis. Although efforts were made to adjust for potential confounders, we cannot rule out the possibility of other covariates that may impact survival outcomes.

Another limitation arises from the exclusive recording of overall survival rather than cancer-specific survival of the NCDB. This data gap makes it difficult to differentiate between deaths specifically caused by AMMLEo and those resulting from other causes. However, overall survival remains a valid and meaningful endpoint, particularly in large-scale studies such as ours, where cancer-specific mortality data may not always be available. Given that AMMLEo is a rare morphologic subtype, it is reasonable to consider overall survival as a key metric for evaluating long-term patient outcomes.

The strength of this study lies in its large sample size, despite the rarity of AMMLEo. Using the NCDB, covering over 70 % of U.S. cancer diagnoses, we note potential inconsistencies as it excludes non-Commission on Cancer-accredited facilities. Despite its retrospective nature and limited data, the findings of this study align with existing literature, supporting the conclusions.

## Conclusion

Several factors associated with worse overall survival in AMMLEo patients were identified. Generally, older African-American patients with comorbidities who did not have private insurance had decreased overall survival. Also, no survival benefit was found for the primary sites (bone marrow and peripheral blood), median household income, and patient sex. These findings are clinically relevant and contribute to AMMLEo comprehension, especially as this rare cancer subtype becomes more accurately defined.

## Disclosure statement

The study protocol was reviewed by the Creighton University Institutional Review Board (IRB# 2001,750–01). The IRB determined that this project does not constitute human subjects research as defined by 45 CFR 46.102(f); consequently, formal informed consent was not required

## Payment/Services

This research did not receive any specific grant from funding agencies in the public, commercial, or not-for-profit sectors.

## Financial relationships

No financial relationships currently or within the prior three years with any organizations that may have interest in the submitted work.

## Authors contribution

All authors contributed to all aspects of the research, including research design, data analysis, and manuscript preparation. Furthermore, all authors reviewed, edited, and approved the final version of the paper.

## Conflicts of interest

The authors declare they have no competing interests.
